# Crescentic glomerulonephritis associated with NK-large granular lymphocytic leukemia: A case report

**DOI:** 10.1097/MD.0000000000043294

**Published:** 2025-07-04

**Authors:** Zengyuan Luo, Zaiyu Wang, Ze Wu, Jieyu Tang, Xiang Ao, Ting Meng, Wei Lin, Rong Tang

**Affiliations:** a Department of Nephrology, Xiangya Hospital, Central South University, Changsha, China; b Department of Pathology, Xiangya Hospital, Central South University, Changsha, China.

**Keywords:** case report, chronic lymphoproliferative disorder of natural killer cells, crescentic glomerulonephritis, NK-large granular lymphocytic leukemia

## Abstract

**Rationale:**

Chronic lymphoproliferative disorder of natural killer cells is a rare heterogeneous indolent hematological disease, characterized by persistent clonal increase of mature NK cells with a typical large granular lymphocyte pattern. Chronic lymphoproliferative disorder of natural killer cells was revised to NK-large granular lymphocytic leukemia (NK-LGLL) in 2022 WHO classification. Renal involvement in NK-LGLL is extremely rare. Here, we report a woman diagnosed with NK-LGLL and nephrotic syndrome.

**Patient concerns:**

A 54-year-old woman had no obvious symptoms except for persistent peripheral lymphocytosis and neutropenia before kidney involvement. Then she presented with nephrotic syndrome, acute kidney injury and Epstein-Barr virus infection.

**Diagnoses:**

Bone marrow displayed clonal increase of mature NK cells with a typical large granular lymphocyte pattern. Renal biopsy showed pauci-immune crescentic glomerulonephritis and renal infiltration by NK-LGLL after exclusion of other diseases. Pathogenic N642H mutation of *STAT5B* was detected by targeted exome sequencing. A319T mutation in *RELN* and R500W mutation in *INTS1* were also identified. Hence, she was diagnosed with crescentic glomerulonephritis associated with NK-LGLL.

**Interventions and outcome:**

We planned to combine methylprednisolone and cyclophosphamide in the treatment of this case. Unfortunately, our patient died of severe cerebral hemorrhage shortly after the diagnosis of NK-LGLL. We had no opportunity to use immunosuppressive drugs for therapy.

**Lessons:**

In short, we report a unique case diagnosed with crescentic glomerulonephritis associated with NK-LGLL, with pathogenic N642H mutation in *STAT5B,* Epstein-Barr virus infection and poor prognosis, different from typical inert type. Close monitoring of renal function is suggested for similar NK-LGLL patients.

## 1. Introduction

Chronic lymphoproliferative disorders of NK cells are rare diseases characterized by persistent expansion of NK cell lymphocytes and neutropenia in peripheral blood, accompanied by an indolent clinical course.^[1]^ Initially classified as a distinct entity in the 2008 WHO classification, chronic lymphoproliferative disorders of NK cells was reclassified as NK-large granular lymphocytic leukemia (NK-LGLL) in the 2022 WHO classification.^[2,3]^ NK-LGLL is categorized as one of the 6 types of mature T-cell and NK-cell leukemia. To date, only a limited number of case reports and small cohort studies on NK-LGLL have been reported.^[4,5]^

Crescentic glomerulonephritis is characterized by the presence of extensive glomerular crescents, and clinically manifests as rapidly progressive glomerulonephritis with acute renal function deterioration.^[6]^ Nephrotic syndrome associated with hematological malignancies has been primarily reported in Hodgkin’s and non-Hodgkin’s lymphomas, and chronic lymphocytic leukemia, while crescentic glomerulonephritis is exceptionally rare in these contexts.^[7]^ Moreover, the presence of glomerulonephritis in NK cell neoplasms is very rare.^[8,9]^ We herein reported a case of nephrotic syndrome and acute kidney injury with pathologically confirmed crescentic glomerulonephritis, as a manifestation of NK-LGLL. To our knowledge, this case is the first report of crescentic glomerulonephritis associated with NK-LGLL.

## 2. Case presentation

A 54-year-old female was admitted to our hospital due to elevated white blood cells for 3 years, edema for 4 months, and worsening for fifteen days. About 3 years ago, her physical examination revealed that white blood cells 12.7 × 10^9^/L, neutrophil 29.22%, and lymphocyte 65.11%, serum creatinine (Scr) 75 μmol/L. Four months prior to admission, she presented with edema and elevated Scr of 178 μmol/L. Fifteen days prior to the admission, she experienced aggravated edema, chest tightness, dyspnea, and elevated Scr (326 μmol/L). She had no personal history of tumor and renal diseases. No similar medical history was found among her family members.

On examination in our hospital, her blood pressure was 158/83 mm Hg, white blood cells 19.9 × 10^9^/L, neutrophil 26.9%, lymphocyte 63.4%, hemoglobin 106 g/L, platelet 239 × 10^9^/L, Scr 324 μmol/L. The serum total protein was 64.0 g/L, albumin levels was 26.4 g/L, and the proteinuria selectivity index was 0.13. The 24 hours urine total protein quantification was 7.3 g, with no hematuria. TBNK showed CD3^+^ 11.41%, CD16^+^CD56^+^ 86.37%, CD19^+^ 1.65%. DNA titer of Epstein-Barr virus (EBV) was 476.3 IU/mL. Serum anti-EBV viral capsid antigen IgG, anti-EBV early antigen IgG, and anti-EBV nuclear antigen IgG were positive. Other indexes including C3, C4, IgG, IgA, IgM, other viral serological markers, antinuclear antibodies, anti-neutrophil cytoplasmic antibody, and anti-glomerular basement membrane antibody were normal. The monoclonal immunoglobulin was negative. Ultrasound showed splenomegaly and multiple lymphadenopathy in the neck, abdomen, and groin. Alternative causes of nephrotic syndrome (e.g., amyloidosis) and other forms of crescentic glomerulonephritis (e.g., ANCA-associated vasculitis, lupus nephritis, anti-GBM disease) were all excluded.

**Figure 1.**
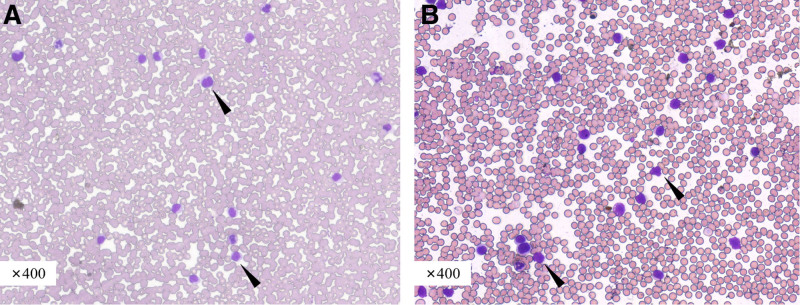
Peripheral blood cytology (A) bone marrow cytology and (B) displayed lymphocytosis in our case. Some lymphocytes showed mild heterogeneity, and intermediate to large in size, as pointed by the arrows.

**Figure 2.**
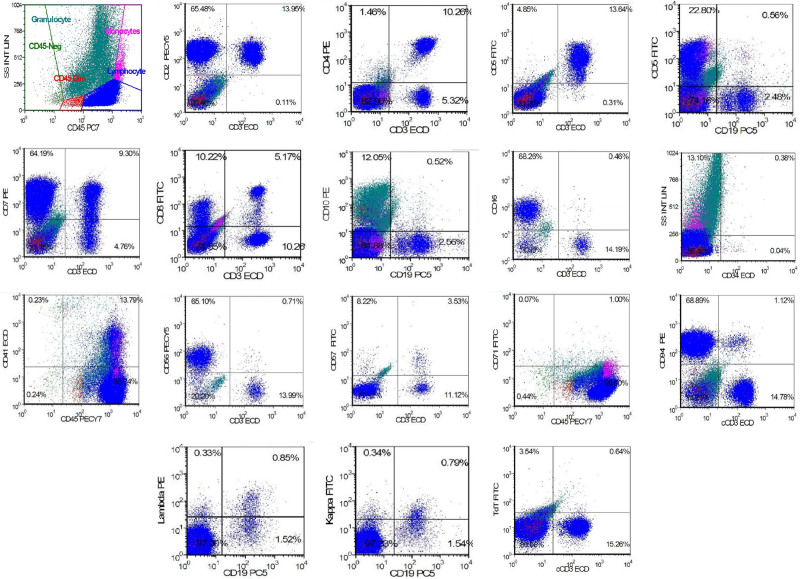
Scatter plots of flow cytometry immunophenotyping (FCI) in bone marrow. Bone marrow cells were stained by flow cytometry with surface markers including CD2, CD3, cCD3, CD4, CD5, CD7, CD8, CD10, CD16, CD19, CD20, CD34, CD41, CD45, CD56, C57, CD71, TdT, Kappa, and Lambda. Abnormal NK cells displayed a CD2^+^, CD3^−^, CD7^+^, CD16^+^, CD56^+^, CD94^+^, CD8^+^, cCD3^−^, TDT^−^, CD4^−^, CD5^−^, CD19^−^, CD20^−^, CD34^−^, and CD57^−^ immunophenotype in bone marrow. ECD = phycoerythrin-texas red conjugate, FCI = flow cytometry immunophenotyping, FITC = fluorescein Isothiocyanate, PE = phycoerythrin, PECY5 = phycoerythrin-cyanine 5, SSINTLI = specific stain intensity, TdT = terminal deoxynucleotidyl transferase.

As shown in Figure 1A, an increase in mature lymphocytes (65%) was displayed in peripheral blood. Notably, bone marrow displayed increased proliferation, granular lineage accounted for 34%, erythroid lineage ratio was 11%, and megakaryocytes were visible, and lymphocytes ratio was increased (Fig. 1B). The aberrant NK cells proportion was 65.34%. Immunophenotypic feature of NK cells in bone marrow showed CD2^+^, CD7^+^, CD16^+^, CD56^+^, CD94^+^, and CD3^−^, cCD3^−^, TdT^−^, CD4^−^, CD5^−^, CD19^−^, CD20^−^, CD34^−^, and CD57^−^. A little NK cells were CD8 positive (Fig. 2). A somatic N642H mutation in exon 16 of *STAT5B* gene was detected in blood. Furthermore, A319T mutation in exon 10 of *RELN* and R500W mutation in exon 11 of *INTS1* which might be may be irrelevant to clinical manifestation were identified by targeted exome sequencing (Table 1).

**Table 1:** Gene mutations identified in our case by targeted exome sequencing.

Gene	Chr.	ExonicFunc	AAChange	SIFT	Mutation frequency (%)
STAT5B	Chr17	Nonsynonymous SNV	NM_012448:exon16:c.A1924C:p.N642H	0.35	20.8
RELN	Chr7	Nonsynonymous SNV	NM_005045:exon10:c.G955A:p.A319T	0.28	18.4
INTS1	Chr17	Nonsynonymous SNV	NM_001080453: exon11:c.C1498T:p.R500W	0	38.9

AAChange = change of amino acid, Chr = chromosome, ExonicFunc = function of amino acid region in exon, INTS1 = integrator complex subunit 1, mutation frequency = the dominant clone allele mutation frequency, RELN = reelin, SNV = single nucleotide variant, SIFT = sorting intolerant from tolerant, SNV = single nucleotide variant, STAT5B = signal transducer and activator of transcription 5B.

**Figure 3.**
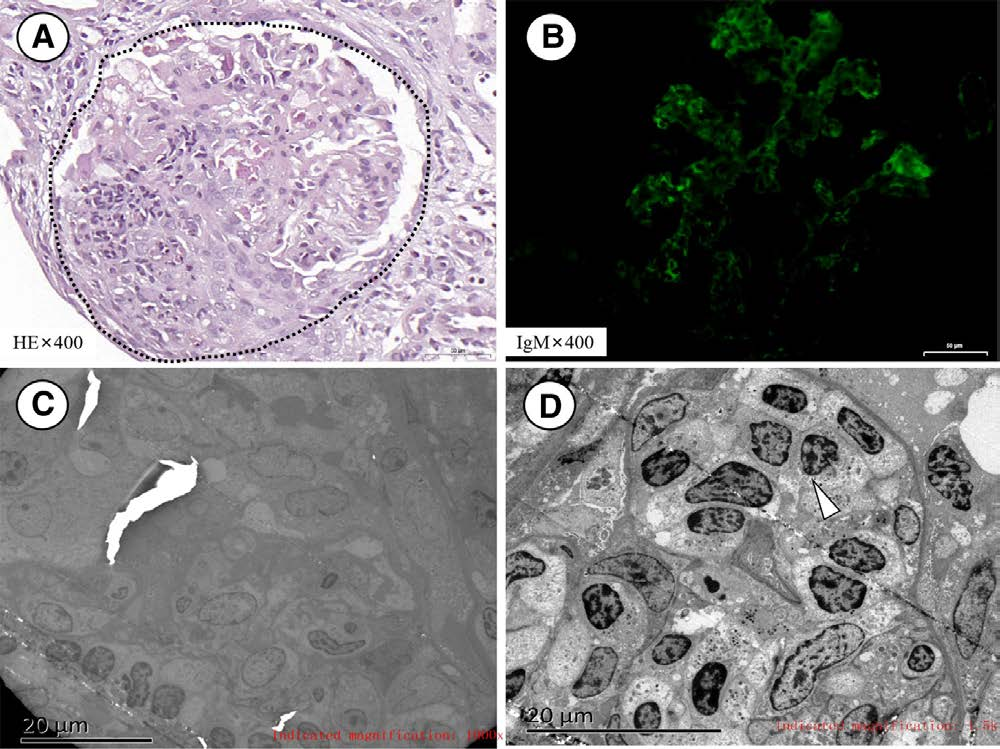
Renal pathological features. (A) Hematoxylin-eosin (HE) staining showed intraglomerular cellular-crescent formation (indicated by circle, ×400); (B) Immunofluorescence showed pauci-immune including negative staining of IgA, IgG, C3, C4, C1q, PLA2R, kappa light chain, and lambda light chain, except IgM 1+ (×400); (C) Electron microscopy (×1000) showed a cellular-crescent formation and also lymphocytes increased in capillaries; and (D) Electron microscopy (×1500) showed that 1 lumen of glomerular capillary was filled with minimally irregular lymphocytes indicated by the white arrowhead. HE = hematoxylin-eosin, IgA = immunoglobulin A, IgG = immunoglobulin G, IgM = immunoglobulin M, PLA2R = phospholipase A2 receptor.

**Figure 4.**
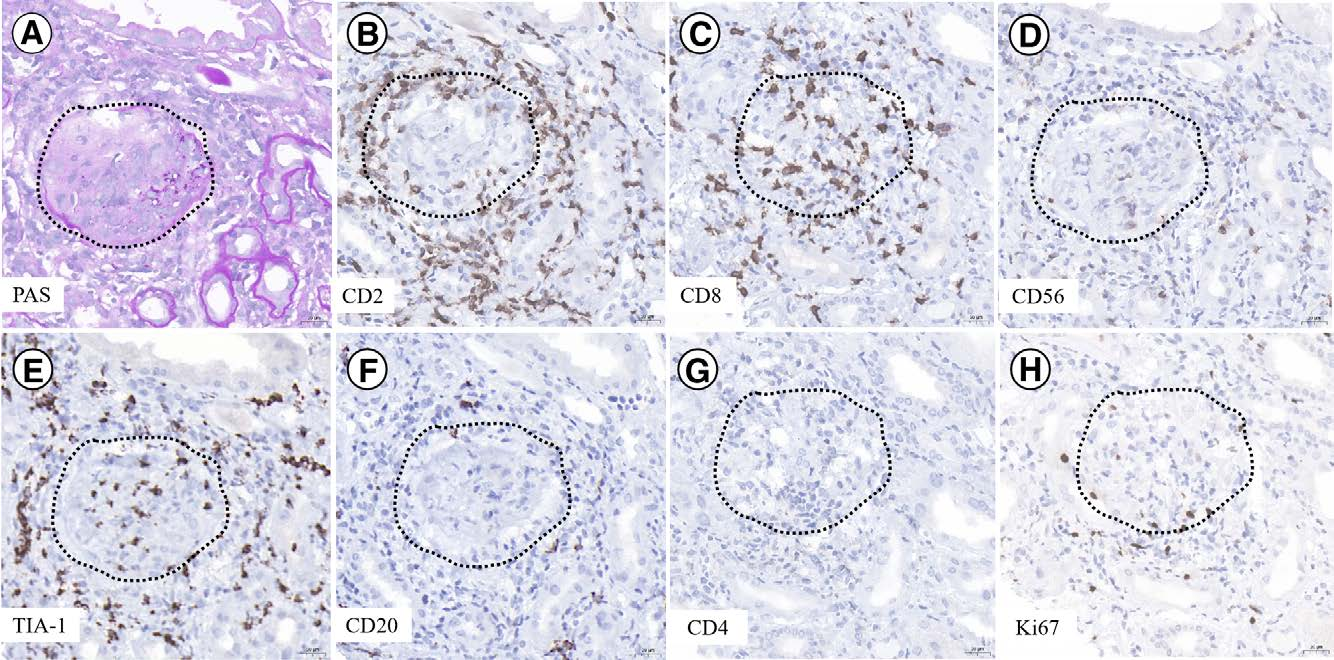
Periodic acid schiff (PAS) and immunohistochemistry staining of kidney. PAS staining (A) showed 2 cellular crescent formation, focal rupture of Bowman’s capsule, medium-sized lymphocytes surrounding the glomerulus, and few within the glomerulus as well. These lymphocytes showed CD2, CD8, CD56, and TIA-1 positivity (B–E), whereas they were CD20 and CD4 negative (F–G) and had a low Ki67 index, 110% (H) (×400). PAS = periodic acid schiff, TIA-1 = T-cell intracellular antigen-1.

Renal biopsy showed pauci-immune crescentic glomerulonephritis (Figs. 3A and 4A). There were 18 glomeruli in total, with 2 global sclerosis (11.11%). 10 crescents (55.56%) were observed (2 cellular crescents, 4 fibro-cellular crescents, and 4 fibrotic crescents), with Bowman’s capsule rupture. Lymphocytes were frequently seen in the lumen of the capillaries. Mild to moderate proliferation of mesangial cells and matrix, focal atrophy and vacuolar degeneration of some tubules were presented. The renal interstitium was scattered with infiltration of inflammatory cells especially lymphocytes, with no obvious vascular lesions. Pauci-immune deposition, except IgM 1+ were shown (Fig. 3B). The medium-sized lymphocytes in and around glomeruli showed CD2^+^, CD3^+^, CD56^+^, CD8^+^, and TIA-1^+^, CD4^−^ and CD20^−^, and low Ki-67 index (Fig. 4B–H). There is a potential inconsistency regarding CD3^+^ in renal tissue versus CD3^−^ in the marrow. In the marrow, CD3 was detected by flow cytometry, which used monoclonal anti-CD3 antibody with high specificity. The CD3 expressed in kidney was detected by immunohistochemistry, which used polyclonal anti-CD3 antibody with higher sensitivity but lower specificity. However, the cells were negative for Epstein-Barr encoding region in situ hybridization. Electron microscopy displayed a cellular- crescent formation and also lymphocytes increased in capillaries, 1 lumen of glomerular capillary was filled with lymphocytes and that the cells are medium to large size, mild atypia, minimally irregular nuclei (Fig. 3C, D).

Combined with the above clinical data, the diagnosis of NK-LGLL was considered. Scr rose to 447 μmol/L after 22 days of hospitalization (the changes in clinical indicators were shown in Table S1, Supplemental Digital Content, https://links.lww.com/MD/P392). Due to pulmonary and urinary tract infection, immunosuppressive drugs have not been used yet. At that time, the patient suddenly felt dizzy, weakness in right limb and altered consciousness. The cerebral computerized tomography showed hemorrhage of the left basal ganglia and the side of the lateral ventricles (4.1 cm × 5.9 cm). An emergency craniotomy was performed. The patient experienced recurrent cerebral hemorrhage 6 hour after surgery, and her family refused another operation. Unfortunately, the patient died 2 days later.

## 3. Discussion

The incidence of NK-LGLL is very low, and crescentic glomerulonephritis associated with NK-LGLL has not been reported due to extremely rare. Here, we described an unusual case of NK-LGLL presented with crescentic glomerulonephritis and acute kidney injury. Intriguingly, N642H mutation of *STAT5B* and EBV infection were also detected in this patient.

NK cell proliferation is deficient in CD3 and T-cell receptor clonality, so it is challenging to differentiate clonal NK-LGLL from reactive NK cell expansion. The diagnosis of NK-LGLL depends on recommended diagnostic criteria including a peripheral persistent LGL expansion (≥0.5 × 10⁹/L and >6 months) with NK immunophenotyping, and the exclusion of other diseases.^[3]^ While the monoclonality of NK-LGLL is difficult to determine, the NK cell phenotype is defined with a typical surface markers CD2^+^, CD3^−^, CD16^+^, and CD56^±^, except for the LGL morphology.^[10,11]^ In our case, the NK cells displayed a CD2^+^, CD3^−^, CD7^+^, CD16^+^, CD56^+^, CD94^+^, CD8^+^, cCD3^−^, TdT^−^, CD4^−^, CD5^−^, CD19^−^, CD20^−^, CD34^−^, and CD57^−^ immunophenotype in bone marrow, which indicated the diagnosis of NK-LGLL.

NK-LGLL is easy to miss or misdiagnose because about half of them are asymptomatic, with very few aggressive form. The major clinical features of NK-LGLL include neutropenia, fatigue and/or B symptoms, autoimmune associated diseases such as rheumatoid arthritis, autoimmune cytopenia, and peripheral neuropathy.^[4,12]^ Our case had no obvious symptoms except for lymphocytosis and neutropenia before the kidney was involved. This case manifested as pauci-immune pattern of crescentic glomerulonephritis, nephrotic syndrome, and EBV infection, with poor prognosis. EBV infection is seldom positively detected in patients with NK-LGLL.^[13]^ Studies have reported the transformation of NK-LGLL with active EBV infection into aggressive NK cell leukemia or lymphoma, and long-term follow-up is necessary in the future.^[14]^ It indicated that EBV infection in NK-LGLL probably played a critical role in the pathogenesis, which might partly explain the aggressive course and poor prognosis of this case.

Recently, studies have indicated that some LGLL patients had *STAT3* and *STAT5* somatic mutations, which could be adopted as molecular and diagnostic markers. These mutations may lead to constitutive phosphorylation of the protein, and activation of downstream target genes in JAK/STAT3 pathway, which can sustain LGL proliferation and survival. Most NK-LGLL patients harbor no *STAT5B* mutation.^[15–17]^ Only few report showed that Y655F or N642H mutation in *STAT5B* have been identified in 2 patient with NK-LGLL respectively.^[18,19]^ Interestingly, a study found that *STAT5B* mutation impaired human NK cell maturation which leads to autoimmunity, recurrent infections, and combined immune deficiency.^[20]^ Pathogenic N642H mutation of *STAT5B* was defined in our patient, which serve as a proof to differentiate from reactive NK cell proliferation. It is suggested that LGLL cases with *STAT5B* mutations possess a more aggressive course, different from typical LGLL with a relatively favorable prognosis.^[16]^ Our case with *STAT5B* mutation manifested as an aggressive variant with poor outcome, indicating aberrant STAT5B signaling underlies the pathogenesis of this rare disease.

At first glance, the simultaneous occurrence of NK-LGLL and crescentic glomerulonephritis appeared to be accidental presence of 2 uncommon disorders. CD2, CD3, CD56, CD8, and TIA-1 were positive in kidneys, and CD4, CD20, CD138 were negative, but with low Ki-67 index, indicating the renal infiltration of NK-LGLL. We observed that the infiltrating NK cells in kidney frequently surrounded the glomeruli, some of which were accompanied by rupture of the Bowman’s capsule, and hypothesized that glomerular crescent formation might be related to the direct destruction of Bowman’s by NK cells. Also, NK-LGLL may affect the kidney through other multiple mechanisms that include direct infiltration, disturbance of immunity, paraneoplastic syndrome, viral infection such as EBV, and immune disorders caused by *STAT5B* mutation.

To date, there is no standard management strategy for patients with NK-LGLL, most patients only need follow-up and observation. The treatment indications for NK-LGLL include cytopenias, associated autoimmune disease, and symptomatic or transfusion dependent anemia.^[4]^ Immunosuppressive therapy remains the common treatment regimens for NK-LGLL including cyclophosphamide, methotrexate, cyclosporine, and steroids. Our case with crescentic glomerulonephritis meeted the treatment indications, and we planed to combine methylprednisolone and cyclophosphamide. Unfortunately, our patient died of severe cerebral hemorrhage shortly after the diagnosis of NK-LGLL. We had no opportunity to use immunosuppressive drugs for therapy. If the patient had treatment commenced, there will be a decrease in NK cells and a reduction in the immune inflammatory response in the kidneys, which could improve the prognosis of patient.

## 4. Conclusion

In brief, we described a unique case diagnosed with nephrotic syndrome and crescentic glomerulonephritis associated with NK-LGLL, distinct from typical indolent subtype. EBV infection and the pathogenic N642H mutation in *STAT5B* were identified in this case, suggesting that STAT5B inhibitors may be a novel therapeutic strategy. We hope this case will enhance understanding of the clinical and pathological spectrum of NK-LGLL. Hence, early diagnosis is crucial to promptly follow patients and initiate appropriate immunosuppressive therapy upon manifestation of clinical symptoms. Closely monitoring of renal function is essential in NK-LGLL patients. Further investigations are required to clarify the mechanisms linking NK-LGLL and crescentic glomerulonephritis.

## Author contributions

**Conceptualization:** Wei Lin, Rong Tang.

**Data curation:** Zengyuan Luo, Zaiyu Wang.

**Investigation:** Zengyuan Luo.

**Methodology:** Zaiyu Wang, Ze Wu.

**Resources:** Jieyu Tang, Ting Meng.

**Supervision:** Xiang Ao.

**Funding acquisition:** Rong Tang.

**Writing – original draft:** Zaiyu Wang, Zengyuan Luo.

**Writing – review & editing:** Zengyuan Luo, Wei Lin, Rong Tang.

## Supplementary Material


