# Supplementary material for: Crescentic glomerulonephritis associated with NK-large granular lymphocytic leukemia: A case report
Source: Medicine (Baltimore). 2025 Jul 4;104(27):e43294. doi: 10.1097/MD.0000000000043294 (PMC12237350; doi:10.1097/MD.0000000000043294)
Supplement: Supplementary file 1 [file medi-104-e43294-s001.docx]

**Supplementary Table 1.** Changes in clinical indicators.

| **Time** | **WBC (×10^9^/L)** | **N (%)** | **L (%)** | **Hb (g/L)** | **Scr (μmol/L)** |
| --- | --- | --- | --- | --- | --- |
| 3 years ago | 12.7 | 29.2 | 65.1 | 108 | 75 |
| 4 months prior to admission | 13.0 | 33.0 | 54.9 | 97 | 178 |
| 15 days prior to the admission | 16.8 | 29.5 | 59.5 | 100 | 326 |
| Admission | 19.9 | 26.9 | 63.4 | 106 | 324 |
| After 22 days of hospitalization. | 18.1 | 31.4 | 59.3 | 104 | 447 |

WBC: white blood cells; N: neutrophil; L: lymphocyte; Hb: hemoglobin; Scr: serum creatinine.
